# Popular Science Monthly

**Published:** 1878-08

**Authors:** 


THE August *Popular Science Monthly* has a varied and attractive table of contents. The number opens with a continuation of Prof. Du Bois-Raymond's address on "Civilization and Science," the special subjects of discussion in this installment being "The Technico-Inductive Period," and "The Dangers which threaten Modern Civilization." The next article is a brief but forcible address, delivered at the Harvey Tricentenary by Prof. Huxley, who, on the strength of his own investigations, gives the most emphatic indorsement to Harvey's title as one of the world's greatest discoverers. The third article in this number is the first of two illustrated papers on the teredo, or ship-worm—the creature which makes such havoc of our marine timber. The author, Dr. E. H. von Baumhauer, brings to his subject the results of a long course of observation and experiment, carried on and completed under the auspices of the Government of Holland. Following this is an interesting article by Mr. G. H. Lewes, author of "Problems of Life and Mind," on "The Dread and Dislike of Science." After showing the wide-spread diffusion of such a sentiment, he traces its origin to the united operation of a variety of causes, especially ignorance of what science really is, and the disinclination of most people to have their cherished beliefs disturbed. Under the title "Man and his Structural Affinities," Prof. A. R. Grote, of Buffalo, Vice-President of the American Association, discusses our relationship with the apes, illustrating the subject with a variety of new and accurate representations of ape anatomy and physiognomy. "Voluntary Motion," by Prof. Payton Spence, M. D., is a paper of great interest, especially to teachers, as it brings out in a very striking way the enormous importance of our inherited aptitudes in the process of education.

"Curious Systems of Notation," by T. F. Brownell; "A New Photographic Process" (illustrated); "Monera, and the Problem of Life," by Dr. Edmund Montgomery; "Composite Portraits," by Francis Galton, F. R. S. (illustrated); "Illustrations of the Logic of Science," VI., by C. S. Peirce; and the third of the series on "Poisons of the Intelligence," by Charles Richet, make up, with a fine portrait and pleasantly written sketch of Mr. T. A. Edison, the body of an entertaining and instructive number of this valuable magazine.

Prof. Youmans opens the editorial department with a strong article on "Religion and Science at Vanderbilt University," in which he sharply criticises the inconsistency and bigotry of that institution in turning out Prof. Winchell. "American Influence in Civilization," and a graceful and appreciative tribute to the memory of Mr. George S. Appleton, recently deceased, complete the Editor's Table. The remaining departments maintain their usual high standard of excellence.

